# Scattering-type Scanning Near-Field Optical Microscopy of Polymer-Coated Gold Nanoparticles

**DOI:** 10.1021/acsomega.2c00410

**Published:** 2022-03-24

**Authors:** Stefan G. Stanciu, Denis E. Tranca, Giulia Zampini, Radu Hristu, George A. Stanciu, Xinzhong Chen, Mengkun Liu, Harald A. Stenmark, Loredana Latterini

**Affiliations:** † Center for Microscopy-Microanalysis and Information Processing, Politehnica University of Bucharest, Bucharest, 060042, Romania; ‡ Department of Chemistry, Biology and Biotechnology, Perugia University, Via Elce di sotto, 8, 06123 Perugia, Italy; § Department of Physics and Astronomy, Stony Brook University, Stony Brook, New York 11794, United States; ∥ National Synchrotron Light Source II, Brookhaven National Laboratory, Upton, New York 11973, United States; ⊥ Department of Molecular Cell Biology, Institute for Cancer Research, Oslo University Hospital, Oslo 0379, Norway

## Abstract

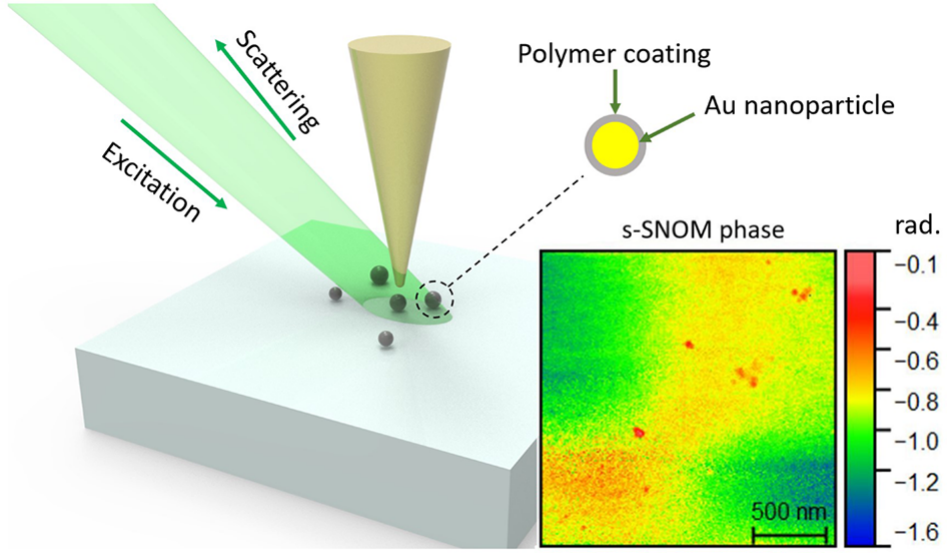

Scattering-type scanning near-field optical microscopy (s-SNOM) has emerged over the past years as a powerful characterization tool that can probe important properties of advanced materials and biological samples in a label-free manner, with spatial resolutions lying in the nanoscale realm. In this work, we explore such usefulness in relationship with an interesting class of materials: polymer-coated gold nanoparticles (NPs). As thoroughly discussed in recent works, the interplay between the Au core and the polymeric shell has been found to be important in many applications devoted to biomedicine. We investigate bare Au NPs next to polystyrenesulfonate (PSS) and poly(diallyldimethylammonium chloride) (PDDA) coated ones under 532 nm laser excitation, an wavelength matching the surface plasmon band of the custom-synthesized nanoparticles. We observe consistent s-SNOM phase signals in the case of bare and shallow-coated Au NPs, whereas for thicker shell instances, these signals fade. For all investigated samples, the s-SNOM amplitude signals were found to be very weak, which may be related to reduced scattering efficiency due to absorption of the incident beam. We consider these observations important, as they may facilitate studies and applications in nanomedicine and nanotechnology where the precise positioning of polymer-coated Au NPs with nanoscale resolution is needed besides their dielectric function and related intrinsic optical properties, which are also quantitatively available with s-SNOM.

## Introduction

Gold nanoparticles (Au NPs) represent one of the most popular types of nanomaterials as they are highly stable and easy to synthesize in various shapes and sizes with reproducible procedures^1^ and, equally importantly, very biocompatible when delivered for therapeutic purposes.^2^ Over the past decades, Au NPs have attracted massive interest as they can interact and be conjugated with various types of molecules, including proteins, nucleic acids, antibodies, enzymes, drugs, and fluorescent dyes, which makes them possess a vast functionalization potential for nanomedicine applications.^3,4^ A particular class of Au NPs consists of those coated with polymers.^5^ As polymers are also highly tailorable and widely used in many biomedical topics, using them in combination with Au NPs significantly augments the number of applications of both materials classes, together with their efficiency. For example, while in general gold nanomaterials are considered safe, studies suggest that such structures can be toxic in some configurations, being prone to be uptaken by kidneys, causing nephrotoxicity.^6^ Given that size, surface charge, chemical composition, and shape are key factors related to the potential toxicity and stability of Au nanomaterials, coating them with polymers in order to tune these properties to values that raise no health hazards represents an interesting solution.^7^ Moreover, given that the elasticity of NPs dictates how these are endocytosed by cells,^8^ controlling this property by coating the Au NPs with soft or rigid polymers^9^ can facilitate various applications that rely on Au NPs uptake or can prevent their internalization when this is not wanted. Other surface properties of the polymer shell have also been found to be important in the context of Au NPs’ cell trafficking.^9^ Exploiting the properties of gold nanostructures to tune the properties of polymer-based materials is also possible. For example, in a past study,^10^ the interrelationship between Au NPs cores and the yield of a fluorescent polymer shell was discussed. Additional aspects on related topics are provided in refs (10 and 11). We also find noteworthy to mention several other previous efforts that built on the synergies existing between Au NPs and polymeric thin films.^12−15^

To improve existing applications of Au NPs and to enable novel ones, a thorough understanding of these materials is required. Many characterization techniques have been used to date in this regard, with optical ones being very important to shed light on various aspects of interest.^16−19^ However, conventional optical microscopies are limited in resolving the properties of Au NPs, as their resolution is limited by the diffraction phenomena to ∼200 nm, depending on the wavelength being used, which is insufficient for assessing nanoscale features of interest. Scattering-type scanning near-field optical microscopy (s-SNOM) represents an emerging optical characterization technique that can help in this regard, offering possibilities for both imaging and spectroscopic assays.^20^ Its working principles rely on a sharp tip that is scanned across the sample’s surface while it is being excited with a focused laser beam. The tip converts the incident radiation into a highly localized and enhanced near field at the tip apex, which modifies both the amplitude and the phase of the scattered light via the near-field interaction with the sample underneath. This process depends on the local dielectric properties of the sample,^21,22^ which can thus be probed with this technique at a resolution dictated only by the dimension of the sharp tip used for scanning and the sensitivity of the detector. Such capabilities are very important for exploring the optical properties of various nanostructures, including various Au-based nanomaterials.^23−28^ Additionally, in previous studies, it was shown that besides using s-SNOM to collect raw near-field optical signals, which are extremely useful, but also quite difficult to interpret, this technique can also be used to quantitatively map with nanoscale spatial resolution the dielectric function and intrinsic optical properties, e.g., refractive index, reflectance, etc.^20,29,30^ Such earlier efforts are currently being extended by means of modern machine learning methods.^31,32^ The attainable resolution in s-SNOM depends on the size and geometry of the probe, with many important applications having been reported for tip sizes ranging between 5 and 150 nm.^22,28,33,34^ In our experiment, we use a Co–Cr tip with a 60 nm radius of curvature in its apex. Also worthy to mention are s-SNOM’s capabilities to lithographically modify materials with superb precision.^35^ Based on these, s-SNOM systems can be regarded as multipurpose platforms for nanoscale manipulation and characterization of advanced functional bio(nano)materials.

While s-SNOM is generally acknowledged as a surface characterization technique, it exhibits nonetheless valuable capabilities for subsurface and 3D imaging. These are very important, considering that subsurface nanostructures cannot be resolved by optical systems with diffraction limited resolution and also cannot be observed by scanning probe or scanning electron microscopy. Although the topic of s-SNOM signals’ attenuation with depth (or by optical obstacles) has been discussed to date in several publications, in relationship with different problems,^36−43^ the body of work performed to date to elucidate such aspects is still limited. Our work aims at extending the current understanding of this problem, focusing on an application that, to the best of our knowledge, has not been addressed before. Namely, we assess the attenuation of s-SNOM phase signals corresponding to a metallic nanoparticle core with the thickness of the surrounding (polymer) shell, when exciting with a laser beam with a wavelength matching the absorption band of the metallic core to promote phase contrast corresponding to the latter. This is a distinct problem compared to the case when the composite nanoparticle is imaged by s-SNOM phase contrast associated with the shell.^44^ Interestingly, we observe very different results compared to previous experiments that evaluated s-SNOM signal attenuation with depth from the perspective of amplitude contrast, instead of phase contrast, which is at the focus of our work. These differences, along with their significance, are discussed in the Results.

To be more specific, in this experiment, we explore s-SNOM’s characterization potential with respect to a set of polymer-coated Au NPs that we carefully synthesized. A custom-modified s-SNOM system was used to acquire amplitude and phase images in the visible frequency range, under illumination with 532 nm, a wavelength falling in the absorption band of the investigated instances, which we demonstrate in detail. In this configuration, with a Co-Cr tip, we obtain strong phase contrast for the bare and shallow-coated Au NPs, whereas the polymer coating seems to attenuate the phase contrast in the case of thick-shell instances. For all instances, the amplitude contrast is minimal, which may be ascribed to reduced scattering efficiency due to absorption of the incident beam by the sample. We discuss as well imaging results obtained with an Au-coated tip, and with the initial Co–Cr one under illumination with an off-resonance wavelength, 1550 nm, none of these two configurations are capable of providing similar s-SNOM phase contrast as observed in the case of the Co–Cr tip illuminated with 532 nm.

We consider our observations to be important, as they may facilitate studies and applications in nanomedicine and nanotechnology where the precise positioning of Au NPs with nanoscale resolution is needed. In this context, we find it worthy to mention that s-SNOM has been demonstrated to date as a very useful tool to image proteins,^45,46^ viruses,^47−49^ prokaryotic and eukaryotic cells,^50−52^ both fixed and living tissues^53^ and various types of nanostructured materials,^34^ including nanoparticles of different kinds,^51,54,55^ providing valuable information based on the sample intrinsic contrast. Such capabilities render s-SNOM as a superb tool for supporting studies that focus on the interaction of advanced materials and biological organisms and structures, at nanoscale. We speculate that some of the important applications exploiting these capabilities could focus on resolving how nanoparticles of different shapes, sizes, and dielectric properties are specifically endocytosed by cells^56,57^ or how their positioning can be correlated with their interference in important cell processes.^58−60^ The results presented here can promote such applications by enabling a better understanding of the attainable s-SNOM contrast for a material class that is not only highly popular in biomedicine^7,61^ but which can also be regarded as a relevant model for other composite nanoparticles comprised of different polymeric-shell metallic core combinations.^62^ Importantly, applications of this kind can also benefit of other opportunities offered by s-SNOM, such as quantitative dielectric function mapping,^29,63^ plasmonic behavior assessment,^64,65^ or the identification of vibrational signatures^66−68^ via spectroscopic variants.

## Methods

### Materials

Gold(III) chloride trihydrate (HAuCl_4_·3H_2_O, >99.9%), trisodium citrate (99%), polystyrenesulfonate (PSS, MW 75 000), and poly(diallyldimethylammonium chloride) (PDDA, MW < 100 000) are all purchased from Merck. Nanopure water (≤15.0 MΩ) from a Millipore Milli-Q gradient system from Merck was used as a solvent.

### Sample Synthesis

The Au NPs were synthesized by means of a multistep approach,^69^ based on the synthesis of small Au NPs which act as “seeds” for the subsequent growth of gold shell, through the control of the Ostwald ripening process. For the synthesis of Au “seeds”, 2.0 mL of sodium citrate solution (60.0 mM) was added to 50.0 mL of water, and the system was heated under magnetic stirring with the presence of a condenser. Once the boiling was reached, 300 μL of HAuCl_4_ solution (25 mM) was quickly injected, and a sudden color change was observed. After 10 min, the seeds were formed, and 630 μL of this colloidal solution was withdrawn for further characterization.

For the growth of the particles, in the same vessel, the temperature of the system was lowered to 90 °C (from then on, the temperature of the system was kept fixed at 90 °C), and 330 μL of sodium citrate solution (60.0 mM) was added. After about 2 min, 300 μL of HAuCl_4_ solution (25 mM) was quickly injected, and the system was left under stirring for 20 min, after which 630 μL was withdrawn for further characterization. This growth procedure was repeated 13 times, which led to obtaining colloidal Au NPs with an absorption peak close to 532 nm, the wavelength of the visible laser line employed in this experiment for s-SNOM imaging. Through this article, we refer to these Au NPs as Au-citr NPs, considering them a type of Au NPs with particular properties as the interactions of the citrate molecules with the gold surface can be different from those that occur in the case of other molecules (citrate establishes ionic interactions that favor Au plasmon damping).^70,71^

Further, the Au-citr NPs synthesized as above-described were coated with polymeric shells consisting of polystyrenesulfonate (PSS) and poly(diallyldimethylammonium chloride) (PDDA), of different thicknesses. The fabrication strategy was based on a layer-by-layer (LbL) process,^10,72^ wherein each polymeric layer was added sequentially. First, 5.0 mL of freshly prepared Au-citr NPs was added dropwise to 5.0 mL of an aqueous PSS solution (0.55 wt %). The system was kept under vigorous magnetic stirring for 1 h. The particles were collected by means of centrifugation (12 000 rpm, 20 min, 10 °C) and dissolved in 5.0 mL of water (this sample was named Au-L1). Afterward, Au-L1 solution was added dropwise to 5.0 mL of an aqueous PDDA solution (0.55 wt %), and the system was kept under vigorous magnetic stirring for 30 min. The particles were collected by means of centrifugation (12 000 rpm, 20 min, 10 °C) and dissolved in 5.0 mL of water (this sample was named Au-L2). By repeating the layer deposition processes, by alternating PSS to PDDA, Au-citr NPs with various polymeric shell thickness were synthesized. The investigations reported in this work were performed on the Au-L3, Au-L5, Au-L7, and Au-L9 samples, where the numerical value in the sample name denotes the number of shell layers (L3: three layers, L5: five layers, etc.). For s-SNOM imaging, the Au-citr NPs and all synthesized Au-L# NPs were deposited on a glass coverslip by spin coating. The coverslip was initially cleaned by immersion in an aqua regia solution (HCl/HNO_3_ 3:1 v/v) for 1 h, followed by several immersions in clean Milli-Q water and finally acetone. Then, 50 μL of Au-citr/Au-L# NPs aqueous suspension was deposited on the center of the coverslip and spin coated at 400 rpm for 10 s, immediately followed by 1500 rpm for 30 s.

### Sample Characterization

The optical properties of the synthesized colloidal samples were first investigated through a Cary 8454 UV–vis diode array spectrophotometer. Transmission electron microscope (TEM) investigations were performed using a Philips 208 transmission electron microscope (TEM) with an 80 kV beam acceleration to assess the size and morphology of the synthesized materials, according to a previously described procedure;^73^ in order to have a statistically significant evaluation at least 120–150 particles for each sample have been analyzed. The particle size analysis was carried by the Nicomp Nano DLS/ZLS Systems (dynamic light scattering particle size analyzer), which allowed the determination of the hydrodynamic diameters of the colloids. All the samples were equilibrated for 10 min at 23 °C, and the runtime was set to 15 min to ensure a good statistic. The NPs were measured in water at 90°, using a red diode laser (635 nm). The scattering data were then analyzed through the Nicomp algorithm.

**Figure 1:**
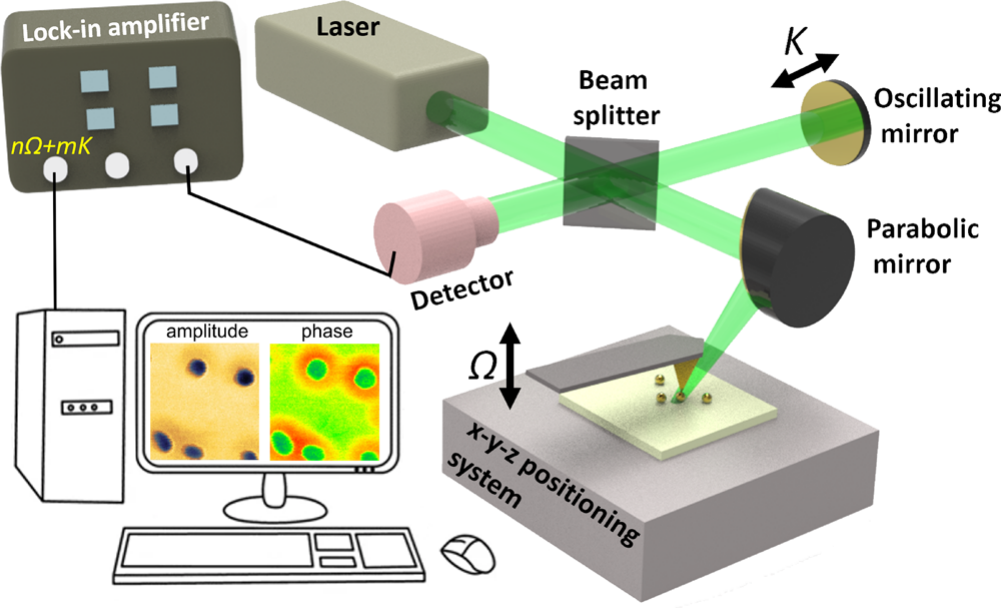
Schematic diagram of a s-SNOM imaging setup operating in pseudo-heterodyne configuration. It relies on a modified Michelson interferometer with one interferometer arm focused onto the tip and the other one reflected off an oscillating reference mirror. The reference beam interferes with the tip-scattered field, and the interference signal carries the near-field information of interest, modulated at nΩ + *mK* frequencies, where Ω and K are the probe and mirror’s oscillation frequencies, respectively. While the tip scans the sample’s *surface*, the near-field amplitude and phase signals are collected using a lock-in amplifier, to result in a nanoscale-resolved s-SNOM amplitude and phase images.

**Figure 2:**
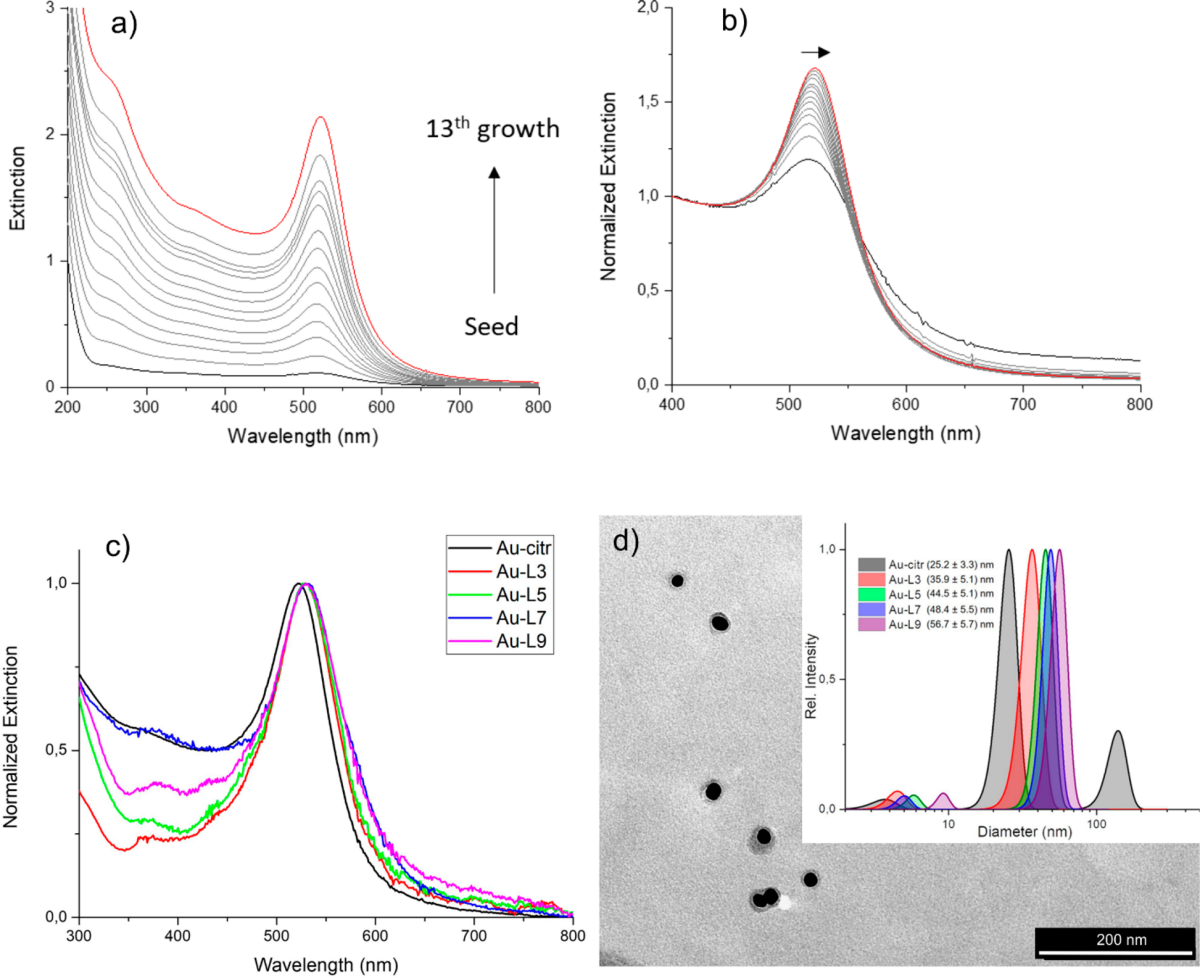
Optical and morphological properties of the synthesized colloidal NPs. (a) Extinction and (b) normalized extinction spectra (at 400 nm) of Au NPs seeds and different growth steps, (c) normalized extinction spectra of bare and polymer-coated Au-citr NPs, (d) TEM image of Au-L3 NPs, inset: DLS measurements of bare and polymer-coated Au-citr NPs, together with the average diameter determined for the samples.

The s-SNOM experiments performed in this work were implemented on a custom-modified neaSNOM (Neaspec GmbH, Germany) system (Figure 1), working in a pseudo-heterodyne configuration. A 532 nm laser beam generated by a Millenia continuous-wave diode-pump solid state laser (Spectra Physics, USA) working in a low power regime was used to excite the polymer-coated Au NPs. According to the performed spectrophotometry investigations, this wavelength matches the absorption band of the Au-citr NPs (Figure 2), thus providing s-SNOM phase contrast for the investigated specimens, as discussed later. Using a neutral filter, the laser power was adjusted <5 mW. The laser beam was injected into a polarization-maintaining single mode fiber by using a 40×/0.65 NA objective lens. The exit of the fiber was connected to a five-degrees adjustable achromatic FiberPort (ThorLabs, USA) collimator used for collimating the beam. A 5× achromatic beam expander (GBE05-B-5X, Thorlabs) was also used to increase the beam diameter to match the maximum numerical aperture of the parabolic mirror of the employed s-SNOM system. Afterward, a pair of two mirrors was used to adjust the laser beam alignment into the s-SNOM unit. For detection, a two-axis adjustable mirror was used to guide the light to the detector (2051-FS, Newport) via a 5 cm focal length lens.

For s-SNOM imaging, a HQ:NSC18/Co-Cr/Al BS (MikroMasch, Bulgaria) cantilever was used, with a radius of curvature < 60 nm. The cantilever had a resonant frequency of 75 kHz and a force constant of 2.8 N/m. Imaging was also attempted with a sharper, Au-coated tip, HQ:NSC16/Cr-Au (MikroMasch, Bulgaria), but this configuration resulted in very weak near-field signals which we hypothesize to be linked with the incident light being absorbed by the Au film coating the tip, resulting in a reduced scattering efficiency.

## Results and Discussion

The aim of this experiment was to assess the capabilities of s-SNOM to image polymer-coated Au NPs, based on phase contrast resulting from the absorption of the excitation beam by the probed sample. As thoroughly discussed in previous works, the phase of near-field signals is related to the complex optical constants using quasi-electrostatic theory,^74^ and importantly, in the landmark work of Stiegler et al.^75^ it was shown that the near-field phase spectra of small particles correlate well with their far-field absorption spectra. Later works^76,77^ had shed more light on the existing correlations occurring between s-SNOM phase signals and the absorption properties of the investigated samples. These previous efforts showed that s-SNOM phase contrast thus provides specificity, as tuning the wavelength of excitation beam to match the absorption properties of a particular element of interest found in an investigated sample leads to images where this element is highlighted. For example, in the recent work of Mészáros et al.,^78^ s-SNOM imaging with illumination at 1660 cm^–1^ (6024 nm) was employed to visualize the protein content (amide I band) and distribution in a number of representative cells.

For the polymer-coated Au NPs discussed in this study, s-SNOM phase contrast could thus arise either from the polymer shell or from the Au-citr NP core. s-SNOM phase contrast arising from polymers has been thoroughly discussed in past works,^66,79−81^ and for this class of materials, mid-IR illumination sources are required. Our interest addresses the distinct problem of probing the phase contrast arising from the Au-citr core, which has a two-fold reason. First, the findings can be generalized for additional composite colloidal nanomaterials based on Au cores covered with shells composed of various other materials.^82^ Second, with correlative imaging approaches based on multimodal systems that combine s-SNOM with other near-field and far-field modalities,^53,83^ the use of excitation sources in the visible or near-IR to serve multiple imaging modalities is more practical and more financially feasible given that other complementary near-field modalities such as tip-enhanced fluorescence, tip-enhanced Raman, tip-enhanced second-harmonic generation, as well as far-field modalities such as diffraction limited and super-resolved techniques based on laser scanning, typically operate under visible and near-infrared excitation. Correlative imaging approaches based on multimodal systems that incorporate various combinations of these aforementioned techniques could be particularly useful in assessing how polymer-coated Au NPs position inside cells, by providing both a well understood biological context, based on fluorescence imaging of the cells at microscale, together with the possibilities for nanoscale localization of Au NPs via label-free s-SNOM phase contrast. Finally, illumination sources in the visible are more widespread and more affordable than mid-IR and IR ones, which also motivates our interest to explore related s-SNOM applications.

The optical and morphological properties of the synthesized NPs are presented in Figure 2. Figure 2a,b illustrates the modifications that occur in the extinction spectra of the Au-citr upon the adopted multistep approach for gold-shell growth (discussed in the Methods). Figure 2c takes a closer look at the (normalized) extinction spectra of the five bare and polymer-coated Au-citr NPs included in this study. A non-normalized spectra is provided in Supporting Information: Figure S1. As can be observed, the absorption/surface plasmon resonance maximum of both Au-citr and Au-L# instances lies close to 532 nm, the wavelength used in this study for s-SNOM illumination. The TEM images of the Au-L3 NPs, as an example (provided in Figure 2d), demonstrate the morphology of the polymer-coated Au-citr NPs, with the Au nucleus being visible in dark black, and the PSS/PDDA shell in light gray; the gray shell presents a slight deformation from spherical shape due to deposition and dryness of the sample on the TEM support and reshaping of the soft-polymer. Our analysis of the TEM images showed that the diameter of metal nuclei presents a Gaussian distribution with a mean size of 20 nm and σ = 2.9 nm, which is slightly lower compared to the average size measured with dynamic light scattering (DLS), 25.2 nm with σ = 3.3 nm, probably due to sampling aspects. In the inset of Figure 2d, we provide information collected with this latter characterization tool on how the size of synthesized NPs varies with the number of shell layers. The size distribution, obtained through analysis of DLS scattering data, provides evidence that the hydrodynamical dimension of the samples progressively increases when increasing the layer deposition steps. It has to be noted that the diameter differences after each deposition step tend to narrow, probably because the polymer corona can pack around the NP surface.

**Figure 3:**
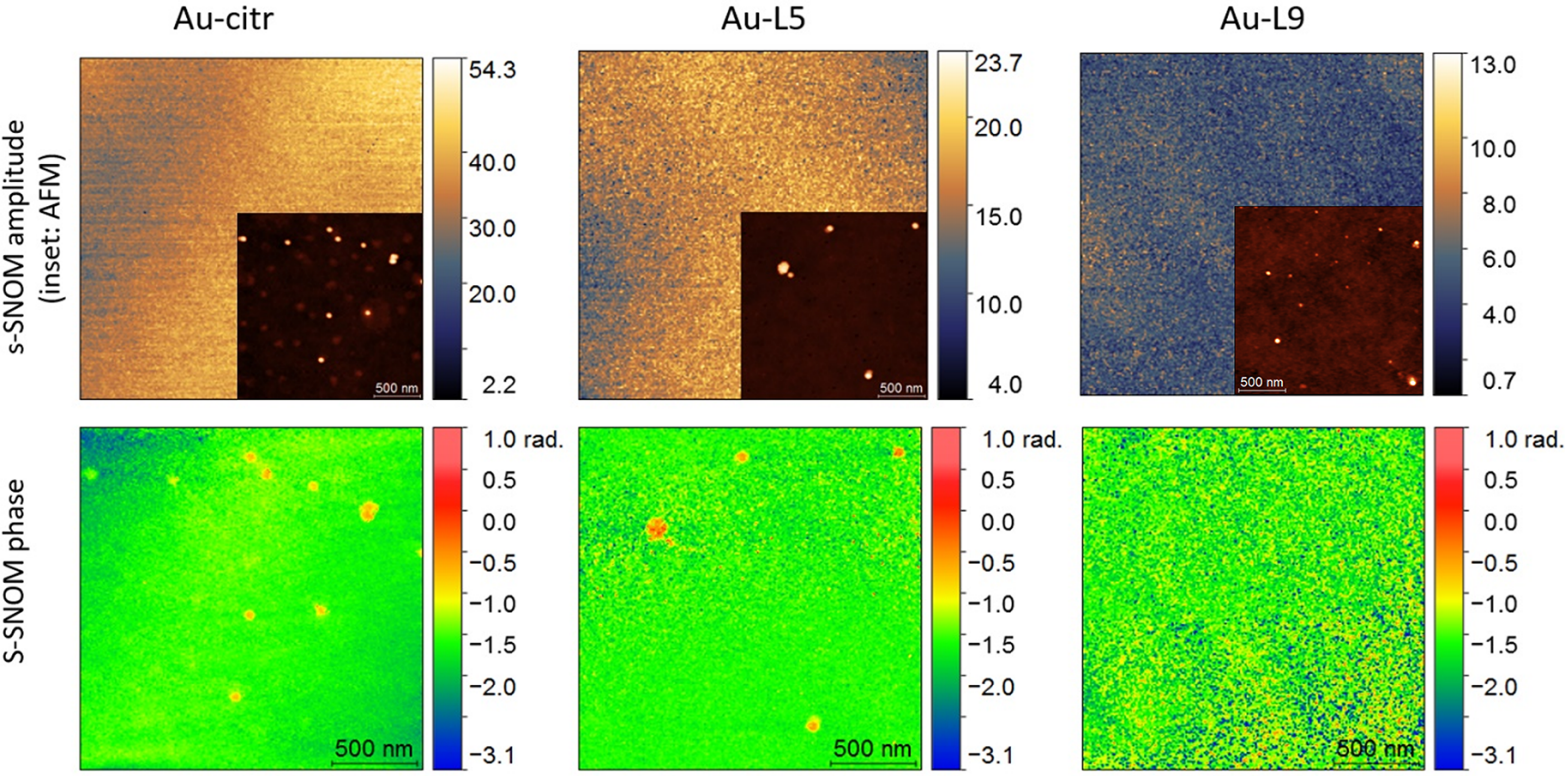
s-SNOM amplitude and phase images of Au-citr, Au-L5, and Au-L9 NPs, collected at the second harmonic of the tapping frequency under 532 nm illumination, with a Co-Cr-coated tip. Insets: correspponding AFM images.

In Figure 3, we present s-SNOM amplitude and phase images for three of the investigated samples Au-citr, Au-L5, and Au-L9, under 532 nm illumination with a Co-Cr-coated tip. Interestingly, for all the samples included in this study, the amplitude signals are very weak, which can be attributed to the absorption of the incident light by the Au-citr NPs, as the fraction of light absorbed by the Au nucleus reduces the fraction of scattered light. On the other hand, the s-SNOM phase signals were found to be consistent for the shallow-coated Au-citr NPs, less visible for Au-L7 (not shown here), and almost entirely missing in the case of Au-L9. In the case of this sample, the low level of the recorded signals can possibly be attributed to an attenuation caused by the polymer shell, the thickest of the studied group. As can be observed, the lateral dimension of the structures visible in the s-SNOM phase images (and also in the acquired AFM images, provided as insets) are higher compared to the NPs’ dimensions probed with DLS and TEM. This can be related to aggregation of the NPs in clusters upon depositing them on the support substrate, and to the size of the tip used for scanning. A combination of these two effects may also apply. s-SNOM amplitude and phase images were also collected at other harmonics of the tapping frequency (second to fifth), and for all cases the remarks mentioned above apply. As discussed also in the Methods, imaging was also attempted with an Au-coated tip, but neither amplitude nor phase signals were available in this configuration, probably due to the absorption of the incident beam by the tip, or due to energy transfer processes occurring between the Au tip and the Au-citr NPs interfering with the scattering efficiency. With the initial Co-Cr tip, we performed imaging as well at an off-resonance wavelength, 1550 nm. This configuration yielded poor contrast in the s-SNOM phase, but a higher signal in s-SNOM amplitude, compared to the case of 532 nm illumination. These latter two situations are depicted in Supporting Information, Figure S2. The results obtained in the three considered configurations also highlight that, for the low height structures here investigated, the topography has a minimal influence on the s-SNOM phase signals.

Further, we discuss additional aspects concerning the intensity of the phase signals observed in the case of all NPs. Given the low intensity of the s-SNOM phase signals observed in the case of Au-L9 (Figure 3), we assume that the polymer shell attenuates the s-SNOM signals. Attenuation of s-SNOM signals corresponding to buried features was also discussed in the recent work of Zhang et al.,^36^ where the authors investigate how the intensity of s-SNOM signals, arising from a patterned Au layer deposited on a Si substrate, modifies with different thicknesses of a PMMA coating placed on top. However, in this work, the authors referred only to s-SNOM amplitude images, which were collected under 10.6 μm excitation, an off-resonance wavelength with respect to both the Au and PMMA. As thoroughly discussed in past works, the phase spectrum of s-SNOM signals is correlated with the sample’s far-field absorption, whereas their amplitude spectrum matches well to the far-field reflectivity spectrum.^54,76,84^ Thus, even though the results presented in this previous work and from our experiment are not straightforward comparable, we find it interesting to note that in this earlier effort s-SNOM was able to sense material contrast (amplitude) between gold and silicon under a PMMA layer with a thickness > 100 nm, whereas in our experiment the s-SNOM phase contrast seems to attenuate more rapidly when imaging polymer-coated Au NPs under 532 nm illumination matching the absorption band of the Au-citr core. This suggests that the problem of s-SNOM signal attenuation with depth is specific to the application at hand and to the considered s-SNOM signal type (e.g., s-SNOM amplitude or phase), aspects that should be carefully considered when designing and implementing an experiment focused on the investigation of subsurface features/elements.

**Figure 4:**
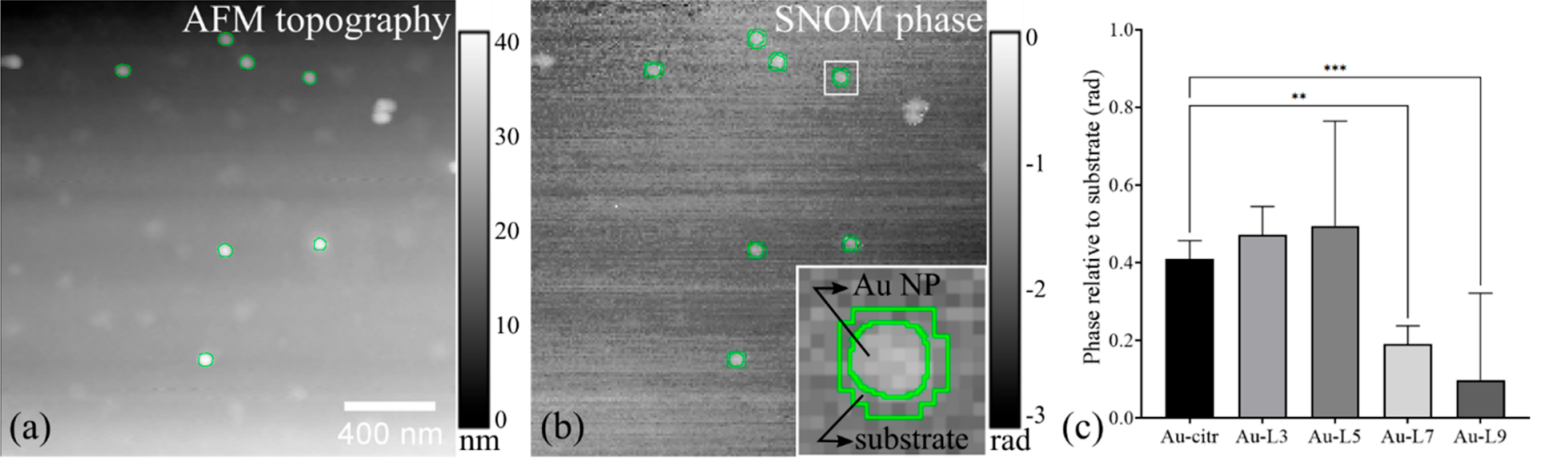
Schematic representation of the analysis protocol. (a) The AFM topography image with the overlaid segmentation. (b) SNOM phase image with the Au NPs segmentation and the substrate mask used to evaluate the substate phase (inset: blow-up view presenting the structure and substrate masks). Both AFM and SNOM images represent raw, unprocessed data. (c) Statistical analysis of the Au NPs samples regarding the SNOM phase change (**P < 0.01, ***P < 0.001). Error bars are 95% CIs.

To shed more light on this, we performed a statistical analysis on the images collected from each sample (two images per sample), testing how the mean phase of the segmented structures varies depending on the polymer shell thickness. In Figure 4, we present the obtained results, together with a representation of the analysis protocol. In this approach, prominent Au NPs according to a height threshold were manually segmented in the AFM topography images using ImageJ.^85^ For each structure of interest, a corresponding doughnut-shaped mask was drawn to cover the surrounding substrate. The width of this irregular shape was manually defined in the range of 1–3 pixels so that the NP structure mask and the surrounding substrate mask have similar areas. The phase of each Au NP was computed as the difference between the average phase in the Au NP mask and the average phase in the substrate mask. This approach is intended to compensate for the phase drift/tilt that usually occurs across the *y* axis when collecting an s-SNOM phase image. Because of this effect, the phase signal values of two identical structures positioned in the upper and lower part of an image frequently differ. This was thus addressed by referring to the phase difference instead of the nominal phase value.

Statistical analysis of s-SNOM phase contrast was performed with Prism 9.1 (GraphPad Software, USA). The one-way ANOVA test was used to determine whether there is a statistical difference between the means of the five independent groups (Au-citr, Au-L3, Au-L5, Au-L7, and Au-L9 s-SNOM phases). We conducted a Dunnett’s test for multiple comparisons to determine exactly which groups are different. Dunnett’s test performed pairwise comparisons between the control Au-citr NPs and four polymer-coated instances. *P* values less than 0.05 were considered statistically significant. The 95% confidence intervals (CIs) of the mean differences between the five groups were also calculated.

The average SNOM phase was computed as above-described for the Au-citr and Au-L3 to Au-L9 NPs identified in AFM topography images. The statistical analysis performed using the one-way ANOVA test on the five NPs groups revealed statistically significant results, which were consolidated by a Dunnett’s test,^86^ indicating a statistically significant decrease in the s-SNOM phase from the control group (Au-citr NPs) to the Au-L7 and Au-L9 instances, which suggests that for both these samples the phase signals are significantly lower compared to the case of the bare Au-citr NPs. The results obtained for the Au-citr NPs versus Au-L3 and Au-citr versus Au-L5 are statistically nonsignificant and thus should be considered with care. The 95% CIs of the differences in each of the two cases (Table 1) contain the null value. Hence, we cannot say with 95% confidence that there is a difference between the two groups. Given that the CIs of the differences also contain positive values, a decrease in the phase signal intensity from Au-citr NPs to Au-L3 and Au-L5 may also be possible. The statistical results for these two comparisons are thus inconclusive. However, on the basis of the 95% CIs (Table 1), and the mean phase relative to the substrate for Au-citr, Au-L3, and Au-L5, we can conclude that the phase values for these three classes lie in similar ranges, which correlate with the acquired s-SNOM phase images that depict consistent signals corresponding to the bare and shallow-coated Au-citr NPs.

**Table 1:** Multiple Comparison Results Provided As the Mean Difference and 95% Confidence Interval of the Difference between Polymer-Coated Au NPs and the Control NPs

Dunnett’s multiple comparisons test	mean difference	95% CI of difference
Au-citr vs Au-L3	–0.06	–0.22–0.10
Au-citr vs Au-L5	–0.08	–0.35–0.18
Au-citr vs Au-L7	0.22	0.06–0.38
Au-citr vs Au-L9	0.31	0.13–0.50

## Conclusions

Au NPs stand among the most intensively used nanomaterials due to their vast potential for functionalization and use in nanomedicine, and nanotechnology in general. A particular class of Au NPs consists of polymer-coated ones, with the interplay between the core and the shell being known to provide important mutual benefits to both materials classes. For example, the polymeric coating can offer various mechanical properties to the Au NPs, which can favor or obstruct their internalization in cells, whereas the plasmonic properties of the Au NPs can be used to tune the optical properties of fluorophore-doped polymer shells. The interplay between the Au core and the polymer coating can be exploited in many other ways, generally being considered a very important chemical engineering tool. In this work, we discuss s-SNOM imaging of Au-citr NPs coated with PSS/PDDA polymeric shells, under illumination in the visible at 532 nm, a wavelength matching the plasmonic band of the Au core. For all samples probed with a Co-Cr tip, we observed weak s-SNOM amplitude signals, which can be ascribed to the absorption of the incident light by Au-citr NPs. Conversely, the s-SNOM phase signals were very consistent for part of the investigated specimens, namely in the case of the bare Au-citr NPs and for the shallow-coated instances, Au-L3 and Au-L5. For the Au NPs coated with thicker shells, Au-L7 and Au-L9, s-SNOM phase signals were found to decrease to the point where for Au-L9 they were no longer visible. Using an alternative Au-coated tip under 532 nm excitation, and the initial Co–Cr under illumination with an off-resonance wavelength, 1550 nm, s-SNOM phase contrast could not be observed for any of the investigated NPs, suggesting a minimal influence of topography on the attainable s-SNOM phase contrast for the case of the investigated structures.

We consider these observations to be important in light of s-SNOM’s capabilities for nanoscale spatial resolution, which represent an important advantage when studying Au NP-related aspects, such as their distribution in doped materials or inside cells. Such latter studies are greatly facilitated by recent efforts that have been focused on s-SNOM imaging of living^51^ and fixed cells,^52^ which have resulted in valuable investigation platforms that can characterize various types of cell structures and processes. We argue that these currently introduced methodologies can be straightforwardly used to assess as well subtle aspects related to the interactions occurring between cells and biomedically functionalized (or hazardous) nanomaterials, to resolve important pending issues such as nanoparticle trafficking by cells.^87^ Hopefully, our experiment will encourage additional work promoting the use of s-SNOM for implementing advanced characterization assays that can resolve properties of polymer-coated Au NPs and also their interaction with biological species, with a specificity and level of detail unavailable to other imaging techniques. Our further efforts will focus on characterizing such polymer-coated Au NPs structures at the single nanoparticle level with ultrasharp tips. Besides experimental work, future efforts based on complex simulations with emerging s-SNOM modeling tools^88^ can also contribute to a better understanding of subsurface s-SNOM imaging.

## Supporting Information Available

Figure S1: Non-normalized extinction spectra of the synthesized colloidal NPs. Figure S2: AFM and s-SNOM images collected on Au-citr NPs. (A) s-SNOM data collected under 532 nm laser beam excitation with an Au-coated tip, (B) s-SNOM data collected under 1550 nm laser beam excitation with a Co–Cr coated tip (PDF)
