# Supplementary material for: Scattering-type Scanning Near-Field Optical Microscopy of Polymer-Coated Gold Nanoparticles
Source: ACS Omega. 2022 Mar 24;7(13):11353–62. doi: 10.1021/acsomega.2c00410 (PMC8992282; doi:10.1021/acsomega.2c00410)
Supplement: Supplementary file 1 — ao2c00410_si_001.pdf [file ao2c00410_si_001.pdf]

Scattering-type Scanning Near-field Optical Microscopy of Polymer-Coated Gold Nanoparticles: Supporting Information

Stefan G. Stanciu[1,*], Denis E. Tranca[1], Giulia Zampini[2], Radu Hristu[1], George A. Stanciu[1], Xinzhong Chen[3], Mengkun Liu[3,4], Harald A. Stenmark[5], and Loredana Latterini[2,*]

Corresponding authors: stefan.g.stanciu@upb.ro; loredana.latterini@unipg.it

[1] Center for Microscopy-Microanalysis and Information Processing, Politehnica University of Bucharest, Bucharest 060042, Romania

[2] Department of Chemistry, Biology and Biotechnology, Perugia University, Via Elce di sotto, 8, 06123 Perugia, Italy

[3] Department of Physics and Astronomy, Stony Brook University, Stony Brook, New York 11794, USA

[4] National Synchrotron Light Source II, Brookhaven National Laboratory, Upton, New York 11973, USA

[5] Department of Molecular Cell Biology, Institute for Cancer Research, Oslo University Hospital, Oslo 0379, Norway.

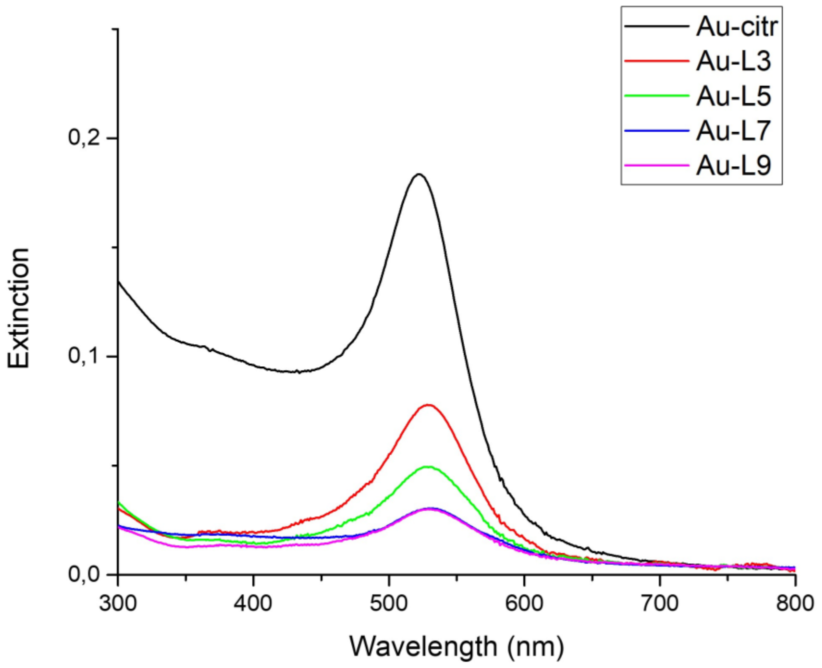

Figure S1: Non-normalized extinction spectra of the synthesized bare and polymer coated NPs.

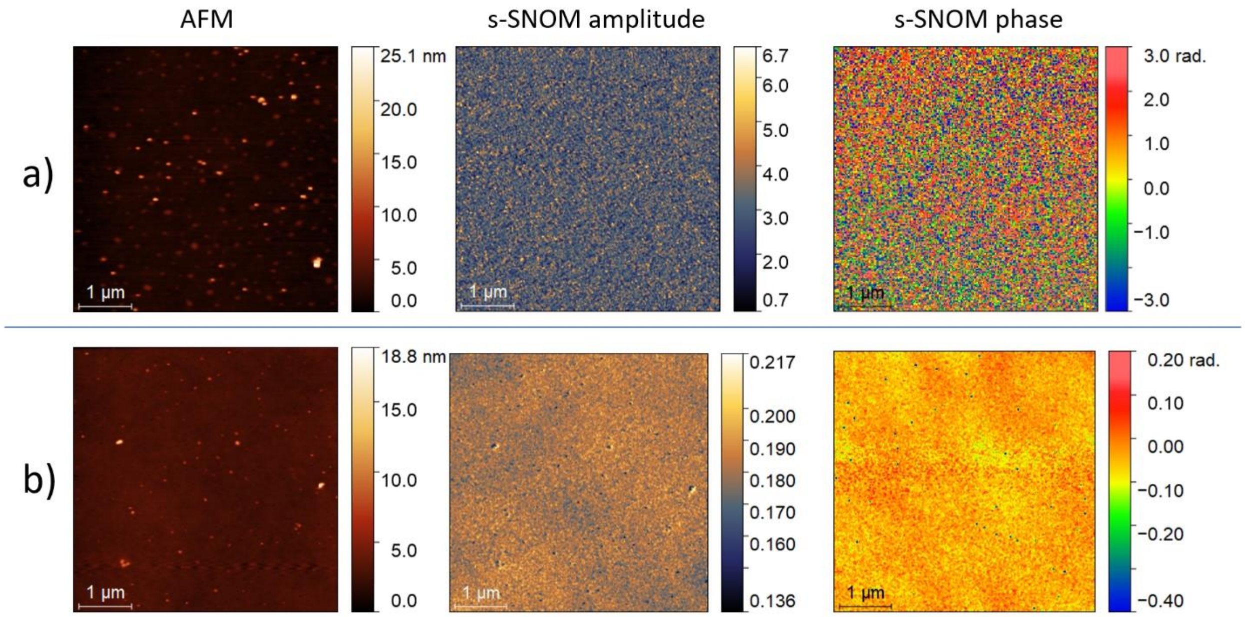

Figure S2: AFM and s-SNOM images collected on Au-citr NPs. A) s-SNOM data collected under 532nm laser beam excitation with an Au coated tip, B) s-SNOM data collected under 1550nm laser beam excitation with a Co-Cr coated tip.
